# Purification and Characterization of a Novel Insecticidal Toxin, μ-sparatoxin-Hv2, from the Venom of the Spider *Heteropoda venatoria*

**DOI:** 10.3390/toxins10060233

**Published:** 2018-06-07

**Authors:** Zhen Xiao, Yunxiao Zhang, Jiao Zeng, Songping Liang, Cheng Tang, Zhonghua Liu

**Affiliations:** The National and Local Joint Engineering Laboratory of Animal Peptide Drug Development, College of Life Sciences, Hunan Normal University, Changsha 410081, China; Zhen-Xiao@outlook.com (Z.X.); Zhangyx@smail.hunnu.edu.cn (Y.Z.); Jiaozeng_@outlook.com (J.Z.); liangsp@hunnu.edu.cn (S.L.)

**Keywords:** spider peptide toxin, bioinsecticide, voltage-gated sodium channels

## Abstract

The venom of the spider *Heteropoda venatoria* produced lethal effect to cockroaches as reported in our previous study, and could be a resource for naturally-occurring insecticides. The present study characterized a novel cockroach voltage-gated sodium channels (Na_V_s) antagonist, μ-sparatoxin-Hv2 (μ-SPRTX-Hv2 for short), from this venom. μ-SPRTX-Hv2 is composed of 37 amino acids and contains six conserved cysteines. We synthesized the toxin by using the chemical synthesis method. The toxin was lethal to cockroaches when intraperitoneally injected, with a LD_50_ value of 2.8 nmol/g of body weight. Electrophysiological data showed that the toxin potently blocked Na_V_s in cockroach dorsal unpaired median (DUM) neurons, with an IC_50_ of 833.7 ± 132.2 nM, but it hardly affected the DUM voltage-gated potassium channels (K_V_s) and the DUM high-voltage-activated calcium channels (HVA Ca_V_s). The toxin also did not affect Na_V_s, HVA Ca_V_s, and Kvs in rat dorsal root ganglion (DRG) neurons, as well as Na_V_ subtypes Na_V_1.3–1.5, Na_V_1.7, and Na_V_1.8. No envenomation symptoms were observed when μ-SPRTX-Hv2 was intraperitoneally injected into mouse at the dose of 7.0 μg/g. In summary, μ-SPRTX-Hv2 is a novel insecticidal toxin from *H. venatoria* venom. It might exhibit its effect by blocking the insect Na_V_s and is a candidate for developing bioinsecticide.

## 1. Introduction

Spiders are the most abundant and successful terrestrial predators, and their venoms are cocktails of toxins including cysteine-rich peptides, neurotoxic proteins, histolytic enzymes, digestive enzymes, linear cytolytic peptides, acylpolyamines, small acids, and amines [1]. Among them, cysteine-rich peptide toxins are rich components of most spider venoms. So far, there are over 47,000 recorded spider species (World Spider Catalog, version 19.0), with some spider venoms containing >1000 different peptides [2]. Therefore, there is a great diversity for spider peptide toxins. Many of these toxins had been proven to be insecticidal and some of them are promising bioinsecticide candidates. According to ArachnoServer 3.0 Spider Toxin Database (www.arachnosever.org) [3], 235 out of a total of 1561 recorded spider peptide toxins were considered to be insecticidal based on either experiment data or their sequence similarity to known insecticidal toxins (accessed on 7 May 2018). The chemical insecticides mainly act on six types of molecular targets in the insect nervous system, including the voltage-gated sodium channels (Na_V_s), glutamate receptors, GABA receptors, nicotinic acetylcholine receptors, acetylcholinesterases, and ryanodine receptors [4,5]. While the insecticidal spider peptide toxins shared with chemical insecticides of Na_V_s as their preferred receptors, they had their own molecular targets in the insect nervous system, including the voltage-gated calcium channels (Ca_V_s), lipid bilayer, calcium-activated potassium channels, presynaptic nerve terminals, and NMDA receptors.

Until now scientists have purified and identified lots of insecticidal spider peptide toxins acting on different molecular targets. For instance, *δ-ct*enitoxin-Pn1a from the venom of Brazilian spider *Phoneutria nigriventer* [6], μ-hexatoxin-Mg1a and μ-hexatoxin-Mg2a, from the venom of Japanese funnel-web spider *Macrothele gigas* [7] slowed the fast inactivation of insect Na_V_s by associating with the DIV S3–4 extracellular loop (site 3 toxins), and δ-amaurobitoxin-PI1a to -PI1d from the venom of the spider *Paracoelotes luctuosus* [8] inhibited the peak currents of insect Na_V_s by binding DII S3–4 extracellular loop (site 4 toxins). The pore region of insect Na_V_s was also a pharmacological binding site for peptide toxins, and the insect-specific insecticidal toxin Sf1a was speculated to be a pore blocker [9]. Most peptide toxins acting on insect Na_V_s were gating modifiers, whose binding sites in insect Na_V_s were distinct from chemical insecticides such as DDT, DDT analogues, and pyrethroids [10]. Thus they might be promising bioinsecticide candidates in terms of managing pests carrying chemical insecticide-resistant Na_V_s mutations, as a previous study showed a spider toxin was active on pyrethroid-resistant strains of peach-potato aphid although the potency was slightly compromised [11]. It is possible to develop insecticides which discriminate pests from beneficial insects by using insect Na_V_s as molecular targets, as inspired by the observation that spider toxins β-Diguetoxin-Dc1a and µ-theraphotoxin-Ae1a even showed selectivity between the highly conserved PaNa_V_1 and BgNa_V_1 channels from two cockroach species [12,13]. ω-hexatoxin-Hv1a [14], ω-hexatoxin-Hv2a [15], and ω-theraphotoxin-Hs2a [16] were insecticidal spider toxins acting on the insect Ca_V_s. Now the toxin ω-hexatoxin-Hv1a was approved as a bioinsecticide by U.S. Environmental Protection Agency (EPA) [17] and was marketed under the trade name of Spear T (www.vestaron.com). Some toxins showed cross activities to the aforementioned bioinsecticides targets, such as the bifunctional toxin μ-NPTX-Nc1a isolated from the venom of the spider *Nephila clavata* and the bifunctional toxins μ/ω-TRTX-Mb1a and μ/ω-TRTX-Mb1b isolated from the venom of the spider *Monocentropus balfouri*. μ-NPTX-Nc1a blocked the cockroach K_V_s and Na_V_s currents, while μ/ω-TRTX-Mb1a and μ/ω-TRTX-Mb1b affected Na_V_s and Ca_V_s currents in the cockroach DUM neurons [18,19]. It was assumed that the bifunctional property of μ-NPTX-Nc1a enhanced its insecticidal potency. There were also some insecticidal spider toxins for which their molecular targets were currently unknown, such as the toxins Ct1a and Ct1b isolated from the venom of the spider *Coremiocnemis tropix* [20], and brachyin, one of the most potent insecticidal toxins, from the venom of the spider *Brachypelma albopilosum* (LD_50_ of 1.02 pm/g and 1.55 pm/g of body weight, to cockroaches and meal beetles, respectively) [21]. Taken together, researches aiming at characterizing potent and eco-friendly insecticidal toxin from spider venoms are still ongoing and will provide us with more and more bioinsecticide candidates.

Our previous study showed that the venom of the spider *H. venatoria* produced a lethal effect when intraperitoneally injected into cockroaches, and the venom potently inhibited the DUM Na_V_s currents [22]. Several peptide toxins have been purified and identified from *H. venatoria* venom as mammalian Ca_V_s (patent number US5627154, 6-May-1997) and K_V_s antagonists [23], while the insecticidal components in this venom were not deeply explored. In the present study, we conducted a full screening of the RP-HPLC purified fractions of *H. venatoria* venom against the DUM Na_V_s and found that a peptide toxin named as μ-SPRTX-Hv2 was the active component. This toxin potently inhibited the DUM Na_V_s and did not affect the currents of DUM Ca_V_s, DUM K_V_s, as well as mammalian Na_V_s. It was lethal to cockroaches, but not mice, when intraperitoneally injected. We suggested μ-SPRTX-Hv2 to be a candidate for developing novel bioinsecticide.

## 2. Results

### 2.1. Characterization of μ-SPRTX-Hv2 as a Cockroach Na_V_s Toxin

**Figure 1:**
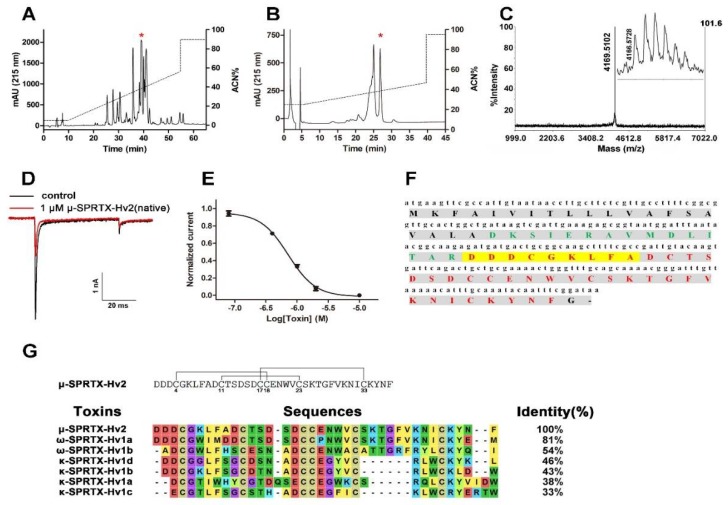
Purification and characterization of μ-SPRTX-Hv2. (**A**) RP-HPLC profile of *H. venatoria* venom, the asterisk indicated the peak containing μ-SPRTX-Hv2; (**B**) μ-SPRTX-Hv2 was purified to homogeneity by analytical RP-HPLC, the asterisk * indicated the μ-SPRTX-Hv2 peak; (**C**) MALDI-TOF MS analysis of purified μ-SPRTX-Hv2, and inset was an enlarged view of the peak; (**D**) μ-SPRTX-Hv2 potently blocked the peak currents of cockroach DUM Na_V_s. Currents were elicited by depolarizations to 0 mV from the holding potential of −90 mV; (**E**) Dose-response curve for μ-SPRTX-Hv2 blocking DUM Na_V_s (*n* = 5); (**F**) Full sequence of μ-SPRTX-Hv2. The signal peptide was shown in black bold, the propeptide was shown in green bold, and the mature peptide was shown in red bold. The sequence determined by Edman degradation was highlighted in yellow; (**G**) Speculated disulfide framework of μ-SPRTX-Hv2 and sequence alignment of μ-SPRTX-Hv2 with toxins characterized in *H. venatoria* venom (the SPRTXs). The residues D and E were shaded in red; M, V, A, L, I, and F were shaded in yellow; G was shaded in fuchsia; W, T, S, Q, and N were shaded in green; C was shaded in olive; Y was shaded in lime; and H,K,R, and P were shaded in teal.

The venom of the spider *H. venatoria* was purified by RP-HPLC (Figure 1A), the eluted fractions were lyophilized and their activities to Na_V_s in acutely dissociated cockroach DUM neurons were tested. This screening analysis confirmed that the fraction with a retention time of 39.4 min was active (Figure 1A, asterisk labeled peak). This fraction was purified to homogeneity by analytical RP-HPLC with a much slower acetonitrile gradient (Figure 1B, asterisk labeled peak). MALDI-TOF MS analysis showed that this peak represented a peptide toxin with the molecular weight of 4169.5102 Da (M + H^+^, Figure 1C). This toxin potently blocked the DUM Na_V_s currents with an IC_50_ of 717.8 ± 40.2 nM (Figure 1D,E, *n* = 5). We determined its partial sequence by Edman degradation, and blasting this sequence in database matched a peptide toxin with the GenBank accession number of AHF45777.1. Its full sequence was shown in Figure 1F. The theoretical molecular weight (4175.63 Da) of the toxin was 7 Da more than that determined by MALDI-TOF MS analysis, indicating that the six cysteines in its sequence formed three disulfide bonds (minus 6 Da), and the C-terminus residue of the peptide derived from the cDNA sequences is glycine, which might be considered as the signal of C-terminal amidation (minus 1 Da). This toxin was described as a secretory peptide in the cDNA library database, and we rationally named the toxin ‘μ-sparatoxin-Hv2’ (‘μ-SPRTX-Hv2’, for short) following the nomenclature rules suggested by King, G. F. et al. [24]. We speculated that μ-SPRTX-Hv2 was an ICK motif toxin based on its “C-C-CC-C-C” cysteine framework (Figure 1G, upper panel). Blasting μ-SPRTX-Hv2 full sequence in NCBI showed that it was the most similar to the Cavs toxin ω-SPRTX-Hv1a (patent number US5627154, 06-MAY-1997) characterized in *H. venatoria* venom (Figure 1G, lower panel), but it showed no significant homology to toxins in other spider venoms. Figure 1G showed the sequence alignment of μ-SPRTX-Hv2 with several known neurotoxins in *H. venatoria* venom by using MEGA7 [25]. We tested the bioactivity of μ-SPRTX-Hv2 to cockroaches and found it produced lethal effect when intraperitoneally injected, the LD_50_ was determined as 3.6 nmol/g of body weight.

### 2.2. μ-SPRTX-Hv2 Synthesis and Activity Assay

**Figure 2:**
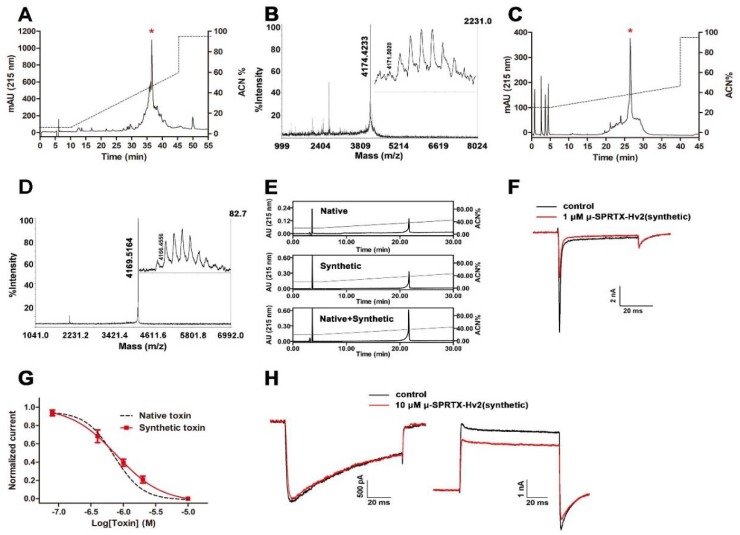
Effects of synthetic μ-SPRTX-Hv2 on cockroach DUM ion channels. (**A**) RP-HPLC purification of crude synthetics of μ-SPRTX-Hv2, and asterisk indicated the peak containing μ-SPRTX-Hv2 linear peptide; (**B**) MALDI-TOF MS analysis of μ-SPRTX-Hv2 linear peptide; (**C**) Analytical RP-HPLC purification of refolded μ-SPRTX-Hv2, and asterisk indicated the correctly-refolded toxin; (**D**) MALDI-TOF MS analysis of the refolded μ-SPRTX-Hv2, and inset was an enlarged view of the peak; (**E**) RP-HPLC co-elution experiment of native and synthetic μ-SPRTX-Hv2; (**F**) Representative traces showed 1 μM synthetic μ-SPRTX-Hv2 potently inhibited the DUM Na_V_s currents. Currents were elicited by 50-ms depolarizations to 0 mV from the holding potential of −90 mV; (**G**) Dose–response curve for synthetic μ-SPRTX-Hv2 blocking DUM Na_V_s. The IC_50_ was determined as 833.7 ± 132.2 nM (*n* = 5). The curve for native toxin was shown in black dashed line; (**H**) Left: representative traces showed 10 μM synthetic μ-SPRTX-Hv2 did not affect the currents of DUM HVA Ca_V_s. Currents were elicited by 100-ms depolarizations to −30 mV from a holding potential of −80 mV (*n* = 4); Right: 10 μM synthetic μ-SPRTX-Hv2 inhibited the DUM K_V_s currents by approximately 15.4 ± 0.1%, currents were elicited by 100-ms depolarizations to +20 mV from a holding potential of −80 mV (*n* = 4).

We chemically synthesized μ-SPRTX-Hv2 and compared its activity with the native toxin. Figure 2A showed the RP-HPLC purification of the crude synthetics, the asterisk labeled peak contained the μ-SPRTX-Hv2 linear peptide. MALDI-TOF MS analysis determined its molecular weight as 4174.4233 Da, which was 1 Da less than the theoretic molecular weight, as the C-terminus of the synthetic peptide was amidated (Figure 2B). This fraction was collected and lyophilized, and refolded as described in the Materials and Methods section. The refolded toxin was subjected to RP-HPLC purification and was eluted at the acetonitrile gradient of approximately 38% (Figure 2C). MALDI-TOF MS analysis confirmed its purity and its molecular weight was consistent with the native toxin (Figure 2D). Co-elution experiment in RP-HPLC showed that the native and the synthetic toxins were co-eluted as a single peak (Figure 2E), suggesting their structural consistency. We tested the insecticidal effect of the synthetic toxin, and its LD_50_ to cockroaches was determined as 2.8 nmol/g of body weight. The representative current traces in Figure 2F showed that the synthetic toxin potently inhibited the cockroach DUM Na_V_s currents. The dose–response curve superimposed with that of the native toxin, with an IC_50_ of 833.7 ± 132.2 nM (Figure 2G, *n* = 5). As the co-elution analysis, the bioactivity and the electrophysiology data all showed that the synthetic and native μ-SPRTX-Hv2 were almost identical, we used the synthetic toxin for further experiments. The toxin did not affect the currents of DUM HVA Ca_V_s even at a concentration of 10 μM (Figure 2H, left, *n* = 4). For K_V_s currents, 10 μM toxin only caused a weak inhibition by approximately 15.4 ± 0.1% (Figure 2H, right, *n* = 4).

### 2.3. μ-SPRTX-Hv2 did not Affect Gating Kinetics of DUM Na_V_s

**Figure 3:**
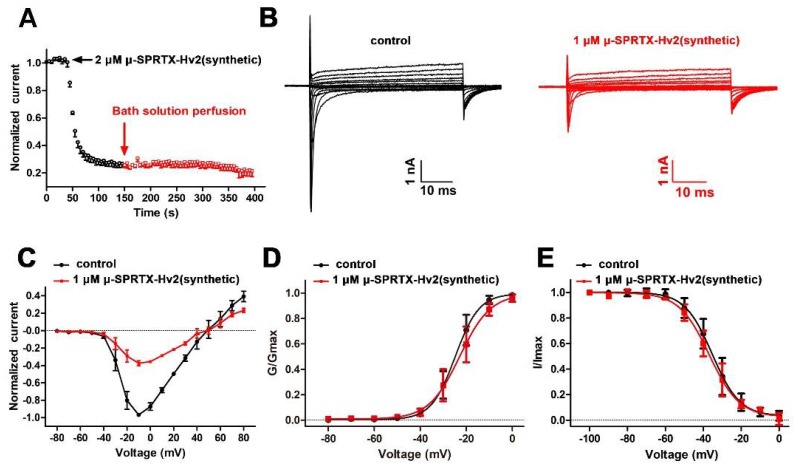
Kinetics of μ-SPRTX-Hv2 interacting with DUM Na_V_s. (**A**) The time course for μ-SPRTX-Hv2 inhibiting DUM Na_V_s currents, and the blocking effect is irreversible. Currents were elicited by 80 consecutive sweeps (each sweep contains a 50-ms depolarization to 0 mV from the holding potential of −90 mV, and the sweep interval was set to be 5 s), normalized to that in the first sweep and plotted as a function of time. The association time constant (τ_on_) was determined as 13.8 ± 1.7 s; (**B**) Representative DUM Na_V_s currents before and after 1 μM μ-SPRTX-Hv2 treatment. Currents were elicited by a cluster of depolarizations from −80 mV to +80 mV, in 10 mV increment, from the holding potential of −90 mV; (**C**) I–V relationships of DUM Na_VS_ before and after 1 μM μ-SPRTX-Hv2 treatment (*n* = 5); (**D**) The G–V curves of DUM Na_V_s before and after 1 μM μ-SPRTX-Hv2 treatment (V_a_ was −24.7 ± 2.6 mV and −23.0 ± 3.7 mV, slope factor was 3.6 ± 0.5 mV and 4.4 ± 0.4 mV, for control and toxin treated channels, respectively; *n* = 5); (**E**) The steady-state inactivation curves of DUM NaVs before and after 1 μM μ-SPRTX-Hv2 treatment (V_h_ was −36.0 ± 4.5 mV and −38.7 ± 4.1 mV, slope factor was −5.3 ± 0.3 mV and −5.4 ± 0.3 mV, for control and toxin treated channels, respectively; *n* = 5). A standard two-pulse protocol was used, in which a 500-ms conditional pulse ranged from −100 mV to 0 mV was followed by a test pulse to −10 mV.

Peptide toxins inhibited the Na_V_s currents either by modifying the activation kinetics or by physically occluding the ion conducting pathway. The toxin μ-SPRTX-Hv2 rapidly inhibited the DUM Na_V_s currents, and the time constant for toxin associating with the channel was determined as 13.8 ± 1.7 s by fitting the decay phase of the trace in Figure 3A. Its effect could not be washed off by bath solution perfusion (Figure 3A), suggesting a very stable binding of the toxin with the channel. To explore the effect of μ-SPRTX-Hv2 on the I–V relationship of DUM Na_V_s, family currents were elicited by serials of 50-ms depolarizations from −80 mV to +80 mV (in 10 mV increment) before and after the application of 1 μM toxin. Figure 3B showed the representative current traces before and after 1 μM μ-SPRTX-Hv2 treatment. The toxin blocked the currents at all voltage tested, but did not affect the initial activation voltage, the peak current voltage and the reversal voltage (Figure 3C, *n* = 5). The steady-state activation curves before and after 1 μM toxin treatment almost superimposed (V_a_ was −24.7 ± 2.6 mV and −23.0 ± 3.7 mV, K_a_ was 3.6 ± 0.5 mV and 4.4 ± 0.4 mV, before and after 1 μM toxin treatment, respectively; Figure 3D, *n* = 5). Furthermore, the toxin did not change the steady-state inactivation of DUM Na_V_s (V_h_ was −36.0 ± 4.5 mV and −38.7 ± 4.1 mV, K_h_ was −5.3 ± 0.3 mV and −5.4 ± 0.3 mV, before and after 1 μM toxin treatment, respectively; Figure 3E, *n* = 5). These data suggested that μ-SPRTX-Hv2 inhibited the peak currents of DUM Na_V_s without affecting the gating kinetics. 

### 2.4. μ-SPRTX-Hv2 did not Act on Mammalian Na_V_s and Ca_V_s

**Figure 4:**
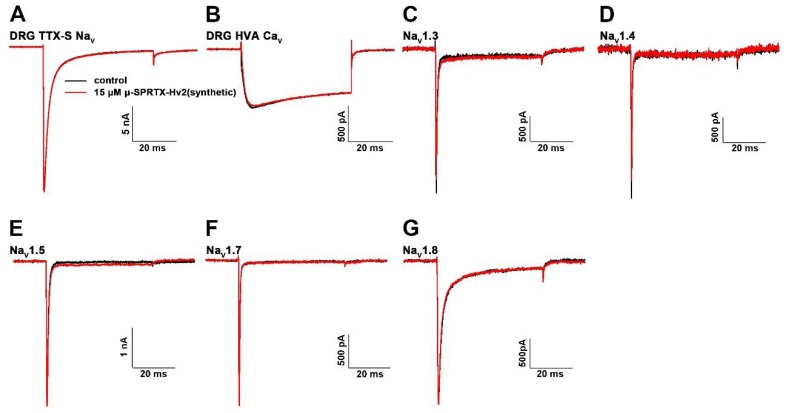
Effects of μ-SPRTX-Hv2 on mammalian ion channels. (**A**,**B**) 15 μM μ-SPRTX-Hv2 did not affect the currents of TTX-S Na_V_s and HVA Ca_V_s in rat DRG neurons (*n* = 5 for each type of currents); (**C**–**G**) Na_V_1.3, Na_V_1.4, Na_V_1.5, and Na_V_1.7 channels were heterologously expressed in HEK293T cells, Na_V_1.8 channel was heterologously expressed in ND7/23 cells. Currents were elicited by depolarizations to +10 mV from a holding potential of −80 mV. These Na_V_ subtypes were resistant to high dose (15 μM) toxin treatment (*n* = 5 for each type of channel).

We tested the toxicity of μ-SPRTX-Hv2 to mouse by intraperitoneally injecting toxin at the dose of 7.0 μg/g, and no obvious envenomation symptoms were observed (*n* = 3). We also tested the activities of μ-SPRTX-Hv2 on mammalian ion channels. The data showed that 15 μM μ-SPRTX-Hv2 did not affect the currents of tetrodotoxin sensitive Na_V_s (TTX-S Na_V_s) and the HVA Ca_V_s in acutely dissociated rat DRG neurons (Figure 4A,B). For heterologously expressed Na_V_ subtypes, 15 μM toxin weakly inhibited the Na_V_1.3 and Na_V_1.4 currents by less than 10%, and did not affect Na_V_1.5, Na_V_1.7, and Na_V_1.8 currents (Figure 4C–G).

## 3. Discussion

The present study has purified and characterized an insecticidal toxin, μ-SPRTX-Hv2, from the venom of the spider *H*. *venatoria*, this toxin possibly functions by blocking the insect Na_V_s. μ-SPRTX-Hv2 did not affect the currents of HVA Ca_V_s and Na_V_s in rat DRG neurons, and it showed no toxic effect when intraperitoneally injected into mouse. We suggested that μ-SPRTX-Hv2 is a promising candidate for developing novel bioinsecticide. Another toxin, ω-SPRTX-Hv1a, isolated from the same spider venom, was reported to be a blocker of Ca_V_s in cerebellar granule cells (Patent number US5627154, 6-May-1997). The μ-SPRTX-Hv2 sequence is highly homologous to that of ω-SPRTX-Hv1a, and their sequence variations mainly located in toxins’ N-terminus and C-terminus (six out of seven amino acid substitutions, Figure 1G). It is interesting to investigate their structural and pharmacological differences in future studies, including testing the activity of ω-SPRTX-Hv1a on insect Na_V_s and that of μ-SPRTX-Hv2 on Ca_V_s in cerebellar granule cells. As a previous study showed even a single amino acid mutation could change both the target selectivity and action mechanism of peptide toxins [26].

The first insect Na_V_ gene (para) was cloned from *Drosophila melanogaster* [27]. After that, lots of studies had cloned Na_V_ genes from many arthropod pests and disease vectors [28], and most insects have only one para-like Na_V_ gene. Na_V_ channels from different insect species have high level of identity (an alignment showed 87–97% identity between several insect species), thus many Na_V_-targeting insecticides had a broad activity across many insects orders [29]. We speculated that μ-SPRTX-Hv2 is also a broad-spectrum insecticide but it is yet to be experimentally determined. In *Blattella germanica*, the Na_V_ gene is BgNa_V_ [30]. However, alternative splicing and RNA editing of BgNa_V_ gene could result in an array of Na_V_s with different pharmacology and gating properties [31,32]. From this point of view, the Navs currents in the isolated DUM neurons might be mediated by several types of BgNa_V_ channels, but they all were blocked by μ-SPRTX-Hv2. There are eight neurotoxin binding sites in Na_V_s, namely site 1–7 and a local anesthetic (LA) binding site [10]. Among them, site 1, site 3, site 4, and site 6 are receptor sites of peptide toxins, with toxins binding to site 3 and site 6 inhibiting the fast inactivation process, and toxin binding to site 1 and site 4 affecting channel activation. The toxin μ-SPRTX-Hv2 in this study inhibited Na_V_s currents without affecting the inactivation process. We speculated that: (1) μ-SPRTX-Hv2 might be a site 1 toxin which functioned by physically blocking the ion conducting pathway, as those of guanidinium toxins (STX and TTX) and μ-Conotoxins acting on mammalian Na_V_s [10]; (2) or, μ-SPRTX-Hv2 bound to the DII S3–4 linker and acted as a gating modifier toxin of insect Na_V_s. Similarly to HWTX-IV acting on Na_V_1.7 channel, μ-SPRTX-Hv2 did not change the steady-state activation curve of insect Na_V_s at physiological depolarizing voltages [33]. The blocking effect of μ-SPRTX-Hv2 to insect Na_V_s was irreversible, as that of δ-hexatoxin-MrIX acting on mammalian Na_V_s [34], suggesting a stable association of the toxin with its binding site. This irreversible binding property of μ-SPRTX-Hv2 actually facilitated its use as an insecticide, and the molecular determinants in insect Na_V_s for binding μ-SPRTX-Hv2 are yet to be elucidated.

It is believed that spiders had an economical use of their venom in preying and defending [35,36]. Our previous study showed each *H. venatoria* spider yielded 2–15 µL of venom and the venom density was 978 µg/µL [22], this translated to a very small volume of venom the spider needed to paralyze the cockroaches (<0.1 µL, the LD_50_ of the venom was 28.2 μg/g of body weight). Thus, it is obvious that μ-SPRTX-Hv2 is not the only insecticidal component in *H. venatoria* venom, as it was only a small fraction of the venom and its LD_50_ to cockroaches was calculated to be 11.7 μg/g of body weight. The spider *H. venatoria* lives on insects, and its venom was optimized by evolution to paralyze or kill the insects. The next study could be to screen the *H. venatoria* venom for insecticidal components acting on other targets, such as Ca_V_s, calcium-activated potassium channels, and so on.

Although an orally active insecticidal peptide toxin, OAIP-1, was isolated from the venom of Australian tarantula *Selenotypus plumipes* [37], most of insecticidal peptide toxins were not active or with diminished activity when taken orally, which hampered their practical use. One strategy to overcome such a barrier was fusing GNA, a mannose-specific lectin from the snowdrop plant, to the insecticidal peptide toxin. As GNA facilitates the transport of the toxin through the insect gut and reaches its action site in the nervous system [38,39]. A previous study showed that the insecticidal fusion protein ω-hexatoxin-Hv1a/GNA had no adverse effects on honeybees [40], which was a public concern of practical use of bioinsecticide in the natural environment. Another strategy was to use recombinant entomopathogen, which was genetically modified to express the insecticidal toxins and showed increased insecticidal potency [41,42]. This approach advanced in systemically producing the toxin in insect after pathogens infection and limiting the off-target effects by using the host selectivity of the pathogen. It is interesting to explore the practical use of μ-SPRTX-Hv2 as bioinsecticide by using these approaches in future studies.

## 4. Materials and Methods

### 4.1. Venom and Toxin Purification

Spiders were captured in corners and eaves of old houses, maintained in terrariums in our laboratory, fed weekly with mealworms and water. A total of approximately 200 spiders were used for venom collection. The venom was collected by an electrical stimulation method as described in our previous study [43], lyophilized and preserved at −80 °C. The crude venom was dissolved in ddH_2_O to a final concentration of 5 mg/mL and immediately subjected to the first round of semi-preparative RP-HPLC purification (C18 column, 10 × 250 mm, 5 μm, Welch Materials Inc., Shanghai, China) using a 45-min linear acetonitrile gradient from 5% to 55% at 3 mL/min flow rate (Hanbon HPLC system equipped with NP7000 serials pump and NU3000 serials UV/VIS detector, Hanbon Sci.&Tech., Huai’an, China). The fraction containing μ-SPRTX-Hv2 was collected, lyophilized, and subjected to the second round of analytical RP-HPLC purification (C18 column, 4.6 × 250 mm, 5 μm, Welch Materials Inc., Shanghai, China) using a 35-min linear acetonitrile gradient from 25% to 46% at 1 mL/min flow rate (Shimadzu HPLC system equipped with LC-20AT pump and SPD-M20A detector, Shimadzu corporation, Kyoto, Japan). The purity of the toxin was tested by MALDI-TOF MS analysis (AB SCIEX TOF/TOF^TM^ 5800 system, Applied Biosystems, Foster City, CA, USA). All mass spectra were acquired in the positive reflectron mode, the laser intensity was 3800. The matrix for MALDI-TOF MS analysis was α-Cyano-4-hydroxycinnamic acid.

### 4.2. Toxin Sequence Determination

The N-terminal sequence of μ-SPRTX-Hv2 was determined by Edman degradation in an automatic protein sequencer (PerkinElmer Life Science Procise 491-A). The *H. venatoria* venom gland cDNA library database was created and submitted to NCBI by Chen. J et al. (College of Bioscience and Biotechnology, Hunan Agricultural University, Changsha, China). The full sequence of μ-SPRTX-Hv2 was determined by blasting the N-terminal sequence against the non-redundant protein sequences database by using the NCBI blast tool (https://blast.ncbi.nlm.nih.gov/Blast.cgi).

### 4.3. Solid-Phase Peptide Synthesis

μ-SPRTX-Hv2 was synthesized by using a Fmoc (*N*-(9-fluorenyl)methoxycarbonyl)/*tert*-butyl strategy and HOBt/TBTU/NMM coupling method [44]. The refolding buffer contains (in mM): 100 NaCl, 5 GSH, 0.5 GSSG, and 100 Tris (pH = 7.4, adjusted with HCl). The linear peptide was diluted with the refolding buffer to a final concentration of 0.01 mg/mL. The solution was stirred slowly at room temperature for 24 h and the refolding reaction was monitored by MALDI-TOF MS analysis. The reaction was terminated by adding TFA to a final concentration of 0.2%, and the reaction mix was subjected to RP-HPLC purification (C18 column, 4.6 × 250 mm, 5 μm, Welch Materials Inc., Shanghai, China) using a 35-min linear acetonitrile gradient from 25% to 46% at 1 mL/min flow rate. The co-elution experiments were performed in Waters 2795 HPLC system equipped with Water 2487 detector (Waters Corporation, Milford, MA, USA) by using a 25-min linear acetonitrile gradient from 20% to 45% at 1 mL/min flow rate (C18 column, 4.6 × 250 mm, 5 μm, Welch Materials Inc., Shanghai, China). 

### 4.4. Bioactivity Assays

Fifty-six cockroaches were randomly divided into eight groups (*n* = 7 in each group). Seven groups were used as experimental groups, to which 10 μL toxin solution (dissolved in saline) was injected between the fourth and fifth sternite, at single dose of 0.29, 0.53, 0.96, 1.74, 3.09, 5.56, or 10 nmol/g for each group. The eighth group was used as experimental control and was injected with 10 μL saline. Lethal effect was observed 24 h after injection. The LD_50_ value was determined by using the improved Karber’s method [45]. For testing the toxicity of μ-SPRTX-Hv2 to mouse, toxin at a single dose of 1.7 nmol/g (7.0 µg/g) was injected intraperitoneally.

### 4.5. Acute Dissociation and Culture of Rat DRG and Insect DUM Neurons

SD rats and C57BL/6 mice (Hunan SJA Laboratory Animal Co., Ltd., Changsha, China) were used according to the guidelines of the National Institutes of Health for care and use of laboratory animals. The experiments were approved by the Animal Care and Use Committee of the College of Medicine, Hunan Normal University. DRG neurons were acutely dissociated from four-weeks-old SD rats and maintained in short-term primary culture as previously described [46]. Briefly, the dissociated dorsal root ganglia were transferred into Dulbecco’s modified Eagle’s medium (DMEM) containing trypsin (0.5 mg/mL, type III) and collagenase (1.0 mg/mL, type IA), then minced with scissor and digested at 37 °C for 30 min. Trypsin inhibitor (1.5 mg/mL, type II-S) was used to terminate the digestion process. The harvested neurons were seeded onto PLL-coated 3.5 cm dishes and cultured for additional 2–4 h, allowing the cells to attach to the dish bottom.

DUM neurons were acutely dissociated from adult cockroaches as previously described [47]. Briefly, the abdominal ganglia were removed from beheaded cockroaches and digested in insect physiological solution (90 mM NaCl, 3.1 mM KCl, 2 mM CaCl_2_, 2 mM MgCl_2_, 140 mM glucose, and 10 mM HEPES (pH = 6.8)) containing papain (20 U/mL) at 37 °C for 15 min. The digestion was terminated with culture medium (200 mM NaCl, 3 mM KCl, 5 mM CaCl_2_, 4 mM MgCl_2_, 50 mM sucrose and 10 mM HEPES, 5% fetal bovine serum, 1% penicillin/streptomycin, pH = 6.8). The harvested DUM neurons were seeded onto PLL-coated dishes and maintained in incubator (5% CO_2_, 28 °C) for 2–3 h before performing patch-clamp analysis. Unless otherwise indicated, chemicals and reagents were products of Sigma-Aldrich (Sigma-Aldrich, St. Louis, MO, USA).

### 4.6. Whole-Cell Currents Recording

Whole-cell recordings of ion channel currents were performed in an EPC-10 patch-clamp platform (HEKA Elektronik, Lambrecht, Germany). The recording pipettes were prepared from glass capillaries (thickness = 0.225 mm) with a PC-10 puller (NARISHIGE, Tokyo, Japan). The serial resistance was controlled to be <10 MΩ, and the voltage errors were compensated by using 80% serial resistance compensation, the speed value for serial resistance compensation was set to be 10 μs. The artificial capacitances were canceled by sequential fast and slow capacitance compensation by using the computer-controlled circuit of the amplifier. All experiments were performed at room temperature. For recording DRG Na_V_s currents, the pipette solution contains (in mM):145 CsCl, 2 MgCl_2_, 10 EGTA, 10 d-glucose, 2 ATP-Na_2_ and 10 HEPES (pH 7.4), and the bath solution contains (in mM):145 NaCl, 2.5 KCl, 1.5 CaCl_2_, 1.2 MgCl_2_, 10 d-glucose, and 10 HEPES (pH = 7.4). For recording DRG Ca_V_s currents, the pipette solution contains (in mM):120 CsCl, 14 phosphocreatine, 10 EGTA, 5 ATP-Mg and 10 HEPES (pH 7.4), and the bath solution contains (in mM): 2 BaCl_2_, 160 tetraethylammonium (TEA)-Cl, 300 nM TTX, 10 d-glucose and 10 HEPES, (pH = 7.4). For recording DUM Na_V_s currents, the pipette solution contains (in mM): 140 CsF, 2 MgCl_2_, 10 EGTA and 10 HEPES (pH = 7.4); and the bath solution contains (in mM): 100 NaCl, 30 TEA-Cl, 2 CaCl_2_, 4 KCl, 10 glucose, 50 choline-Cl, 1 CdCl_2_, 1 4-AP, and 10 HEPES (pH = 7.4). For recording DUM Ca_V_ currents, the pipette solution contains (in mM): 10 Na-acetate, 110 CsCl, 50 TEA-Br, 2 ATP-Na_2_, 0.5 CaCl_2_, 10 EGTA, 10 HEPES (pH = 7.4); and the bath solution contains (in mM): 140 Na acetate, 30 TEA-Br, 3 BaCl_2_, 300 nM TTX, 10 HEPES (pH = 7.4). For recording DUM K_V_s currents, the pipette solution contains (in mM): 135 K-gluconate, 25 KF, 9 NaCl, 0.1 CaCl_2_, 1 MgCl_2_, 1 EGTA, 3 ATP-Na_2_, and 10 HEPES (pH = 7.4); and the bath solution contains (in mM): 200 NaCl, 50 K gluconate, 5 CaCl_2_·2H_2_O, 4 MgCl_2_·6H_2_O, 300 nM TTX, 10 d-glucose, and 10 HEPES (pH = 7.4). For Na_V_ subtype currents recording, hNa_V_1.3 (with 97.18% identity to rNa_V_1.3), rNa_V_1.4, hNa_V_1.5 (with 94.01% identity to rNa_V_1.5), hNa_V_1.7 (with 92.05% identity to rNa_V_1.7), or rNa_V_1.8 cloned in pCDNA3.1 plasmid was co-transfected with pEGFP-N1 plasmid into HEK293T or ND7/23 cells, respectively, by using lipofectamine 2000 following the manufacturer’s instructions. The pipette solution contains (in mM): 140 CsCl, 10 NaCl, 1 EGTA, 2 ATP-Mg, and 20 HEPES (pH = 7.4); and the bath solution contains (in mM):140 NaCl, 2 CaCl_2_, 1 MgCl_2_, 5 KCl, 20 HEPES, and 10 glucose (pH = 7.4).

The electrophysiological data were acquired by using the Patch-Master software. Data were analyzed by using the software Sigma Plot 10.0, Origin 8, and Graphpad Prism 5.01 (GraphPad Software, La Jolla, CA, USA, 2007). The G-V and SSI curves were fitted by a Boltzmann equation: y = y_steady_ + (y_(0)_ − y_steady_)/(1 + exp[(V − V_1/2_)/K]), where V_1/2_, V and K represented the midpoint voltage of kinetics, the test voltage, and the slope factor, respectively. The dose–response curves were fitted by a Hill equation to estimate the potency (IC_50_) of the toxin. The toxin-channel association time constant (τ_on_ value) in Figure 3A was calculated by fitting the decay phase of the trace with the one phase decay equation: y = (y_(0)_ − y_steady_) × exp(−k × x) + y_steady_, in Graphpad Prism 5.01.

### 4.7. Data Analysis

Data were presented as MEAN ± SEM, *n* was presented as the number of separate experimental cells.
